# Supplementary material for: Regional BOLD variability reflects microstructural maturation and neuronal ensheathment in the preterm infant cortex
Source: Nat Commun. 2026 Apr 9;17:4849. doi: 10.1038/s41467-026-71415-x (PMC13222875; doi:10.1038/s41467-026-71415-x)
Supplement: Supplementary file 1 — Supplementary Information [file 41467_2026_71415_MOESM1_ESM.pdf]

Supplementary material

Supplementary Figures

Figure S1. Longitudinal BOLD SD, ALFF and fALFF changes from 33 to 40 weeks in very preterm infants (n=31). Bar plots illustrate the group-averaged BOLD SD, ALFF and fALFF longitudinal changes in VPT infants from 33 wGA to TEA across RSNs, with each metric scaled to its own largest absolute mean regional change. Embedded boxplots show the distribution of individual subject changes within each RSN for each metric, with individual values scaled to the largest absolute subject-level change observed across all RSNs for that metric. The centre line of each boxplot represents the median (50th percentile), the box bounds indicate the 25th–75th percentiles, and the whiskers represent the minimum and maximum values within 1.5×IQR. Regional measurements were derived from the same MRI scans for each participant. Significant changes are indicated by asterisks ("*"p<0.05, "**"p<0.01, "***"p<0.001, after FDR correction). PCUN= precuneus, PCC = posterior cingulate cortex, SSM = sensorimotor, VIS = visual, AUD = auditory, THAL = thalamus, PFC = prefrontal cortex. Source data are provided as a Source Data file.

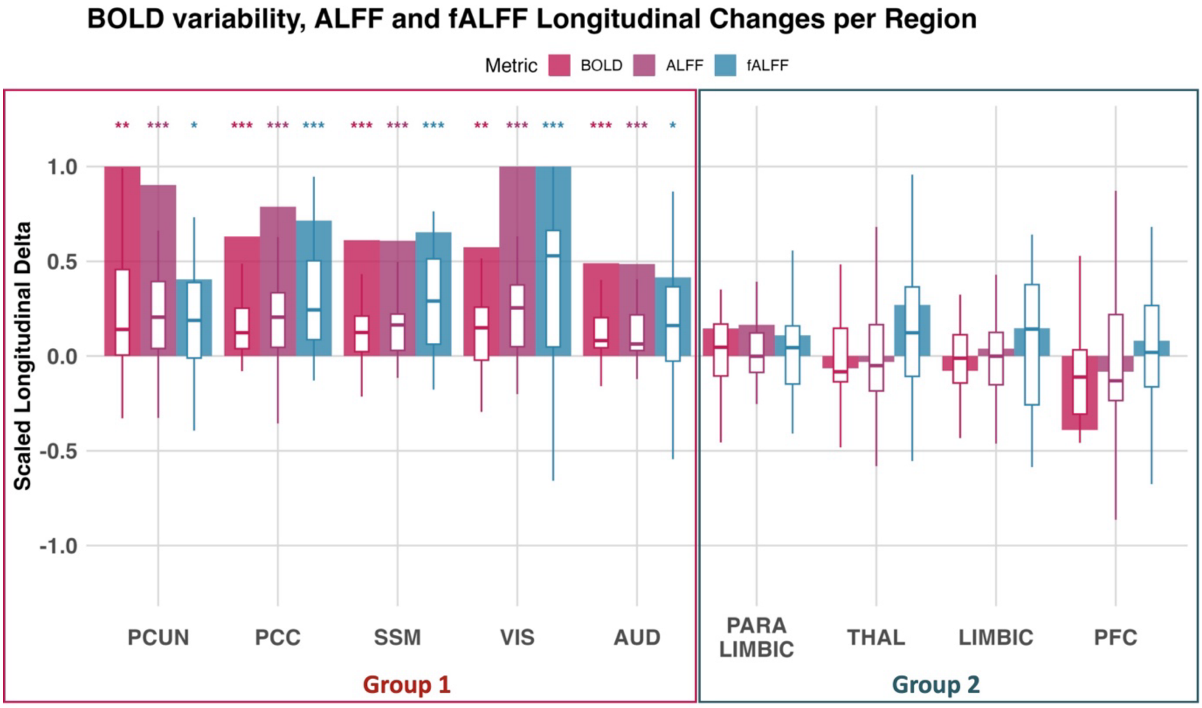

Figure S2. Longitudinal changes in very premature infants' BOLD variability (n=31) and microstructural diffusivities (n=39), from 33- to 40-wGA. Boxplots show the distribution of individual subject delta changes within each RSN for each metric. The centre line of each boxplot represents the median (50th percentile), the box bounds indicate the 25th–75th percentiles, and the whiskers represent the minimum and maximum values within 1.5×IQR. Regional measurements were derived from the same MRI scans for each participant. a) BOLD SD; b-e) SMT metrics, including intrinsic diffusivity (diff), extra-neurite mean diffusivity (extraMD), extra-neurite transverse diffusivity (extraTrans) and intra-neurite volume fraction (intra); f-g) DTI metrics, including mean diffusivity (MD) and fractional anisotropy (FA); h-j) DKI metrics, including mean (MK), radial (RK) and axial kurtosis (AK). Source data are provided as a Source Data file.

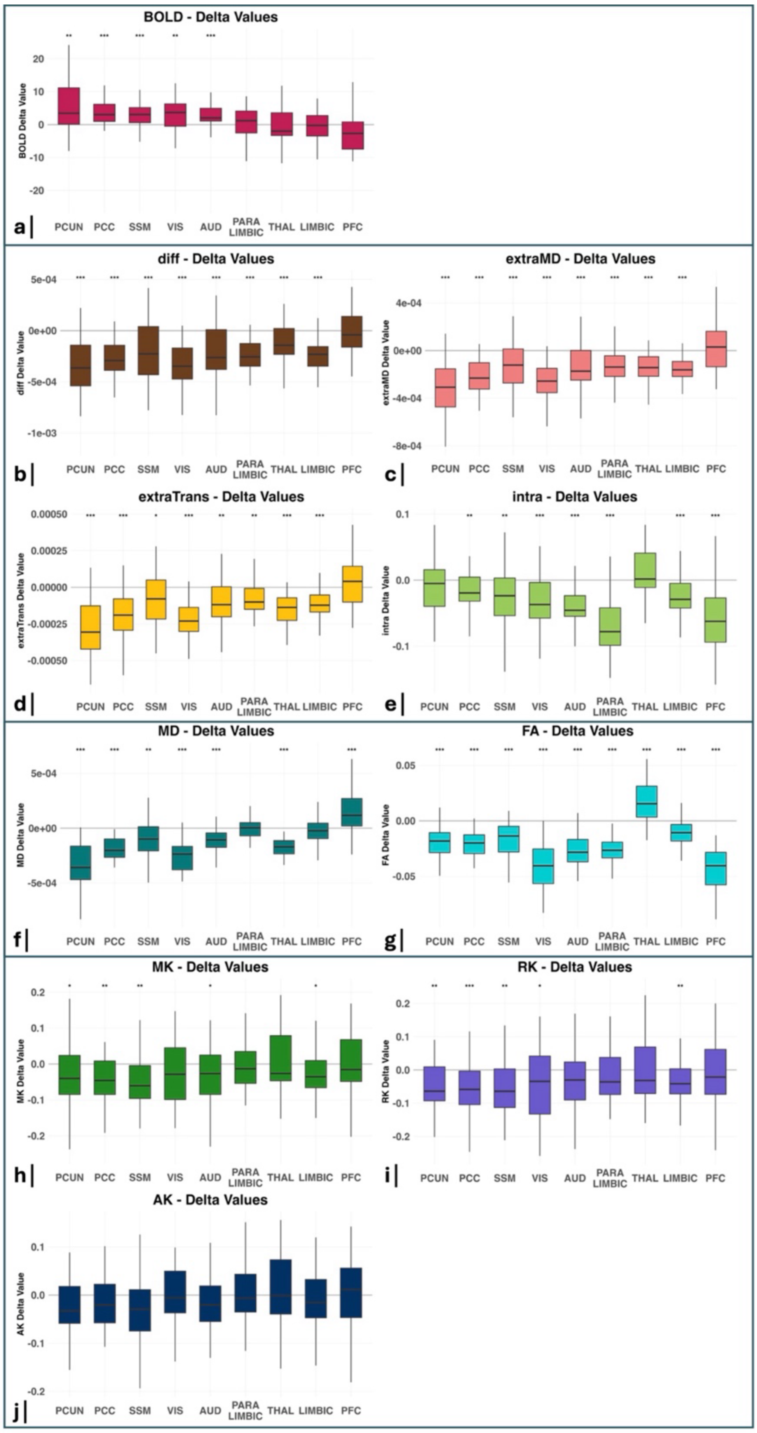

Figure S3. Results from the consensus clustering of the combined 9 distinct diffusion microstructural measures delta changes over time. a) Elbow method showing an inflection point at K = 3, suggesting an optimal clustering solution with three clusters. b) Heatmap depicting the regional maturational patterns from 33- to 40-wGA, with rows and columns representing the RSNs and color intensity in each cell reflecting the composite changes over time of the microstructural metrics. c) Composition of the identified 3 regional clusters of microstructural delta changes (Cluster 1: Thalamus, Cluster 2: PCC, Visual, PCUN, SSM, Auditory, Limbic; Cluster 3: PFC, Paralimbic). d) Brain plots illustrating the 3 distinct regional clusters depicted by the consensus clustering analysis, color-coded according to regional cluster (red = cluster 1, orange = cluster 2, blue = cluster 3). Source data are provided as a Source Data file.

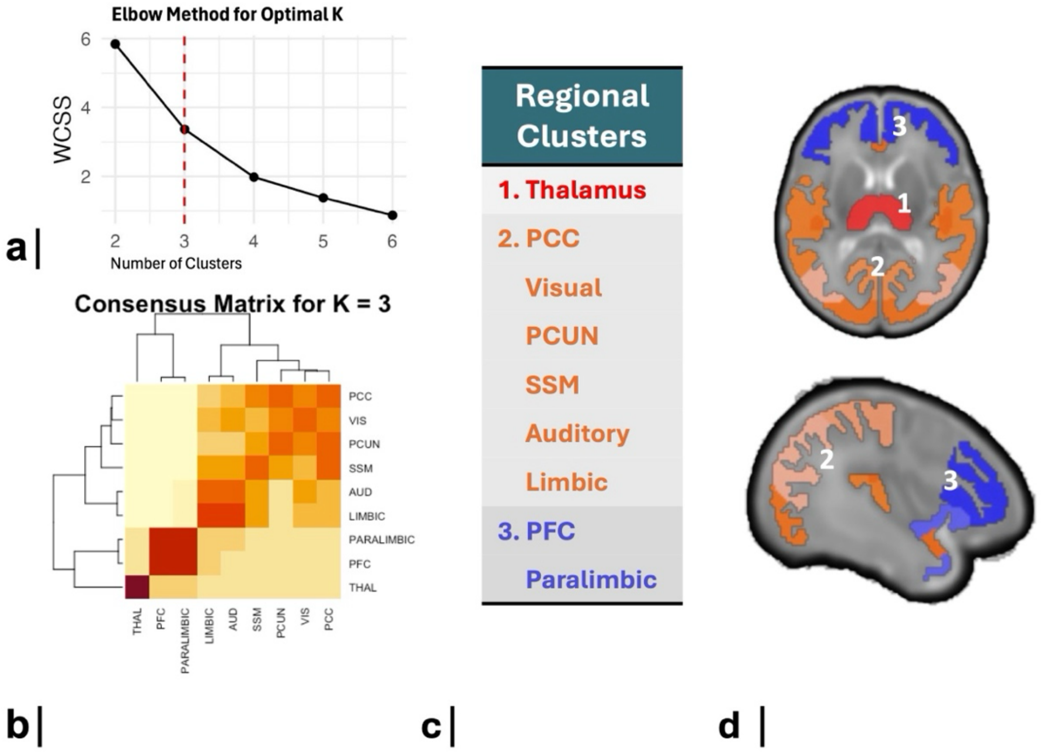

To better understand the spatio-temporal patterns of cortical microstructural maturation across the various RSNs, occurring from 33- to 40-wGA, we have combined the longitudinal changes of all the diffusion microstructural measures across the different dMRI models (DTI, DKI and SMT) and performed a consensus clustering analysis. Microstructural diffusivities were clustered using ConsensusClusterPlus function (k-means base algorithm and Euclidean distance) in R (4.4.1), to identify stable clusters of regional diffusivities. The optimal number of clusters was determined using the elbow method, based on the within-cluster sum of squares (WCSS), indicating an optimal number of clusters at K = 3.

Three distinct regional microstructural maturational patterns were identified, aligning with the central-to-peripheral and posterior-to-anterior known gradients in brain maturation, in agreement with the brain myelination order of Kinney (Kinney et al., 1988). Microstructural cluster 2 aligns with BOLD SD group 1, while Microstructural cluster 3 is more similar to BOLD SD group 2. The thalamus emerged as a separate cluster, reflecting its distinct microstructural profile. This microstructural spatial distribution, although not perfectly identical, is similar to the regional patterns identified by BOLD variability changes, highlighting that the primary sensory-motor and proto-DMN regions exhibit both functional and microstructural changes distinct from those of other brain regions.

Figure S4. BOLD variability (a), ALFF (b) and fALFF (c) differences, between FT (n=19) and VPT (n=31) infants at TEA. Boxplots represent the distribution of individual subject values (FT in blue, VPT in coral) within each network, illustrating inter-subject variability. Bar plots illustrate the average group difference (FT-VPT) for each network. The centre line of each boxplot represents the median (50th percentile), the box bounds indicate the 25th–75th percentiles, and the whiskers represent the minimum and maximum values within 1.5×IQR. Regional measurements were derived from the same MRI scans for each participant. Significant changes are indicated by asterisks ("*"p<0.05, "**"p<0.01, "***"p<0.001, after FDR correction). PCUN= precuneus, PCC = posterior cingulate cortex, SSM = sensorimotor, VIS = visual, AUD = auditory, THAL = thalamus, PFC = prefrontal cortex. VPT = very preterm infants. FT = full-term infants. TEA = term-equivalent age. Source data are provided as a Source Data file.

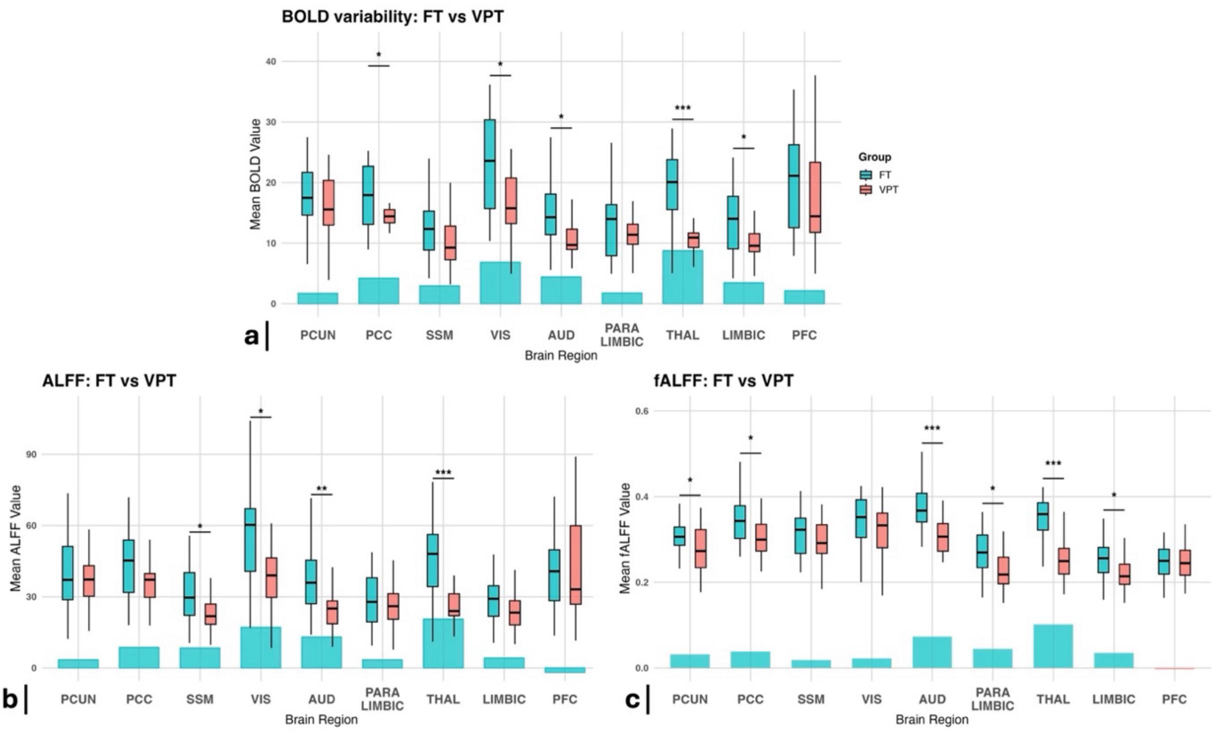

Figure S5. Flow chart of participant selection

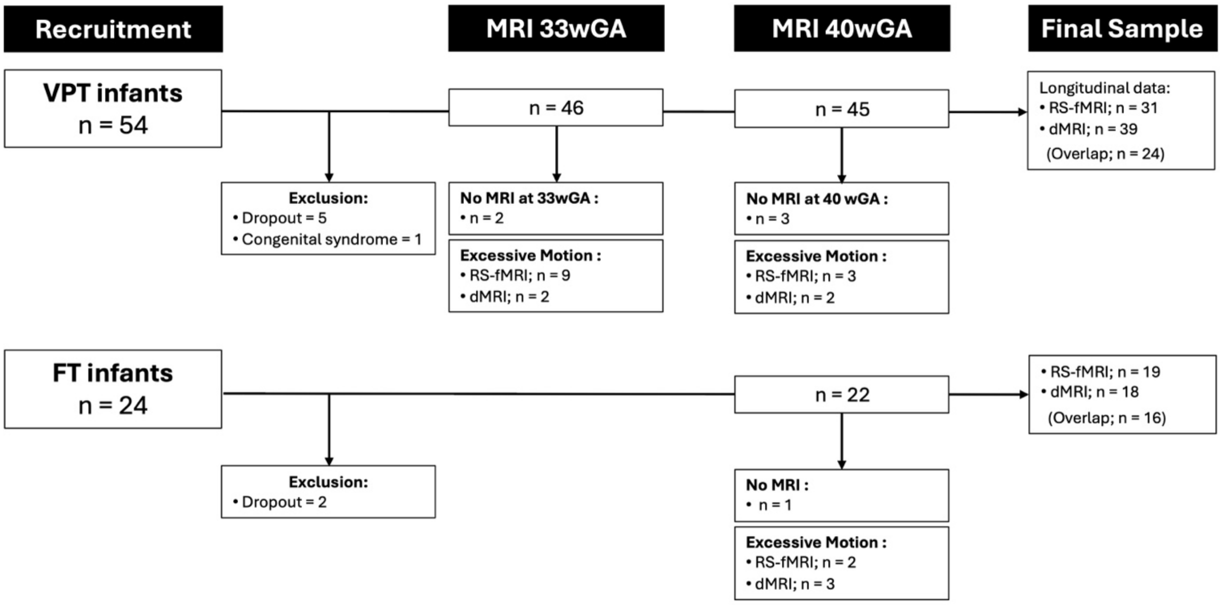

Figure S6. Components obtained from the data-driven ICA group analysis

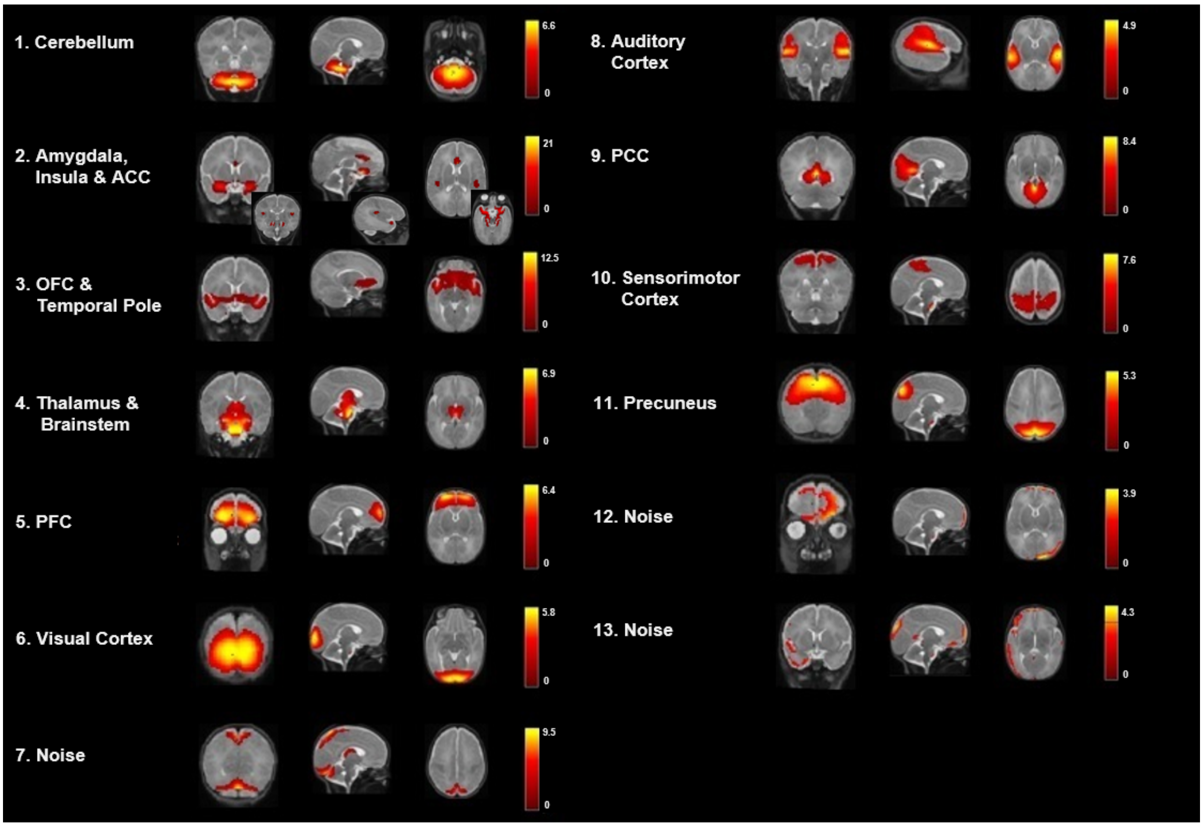

Supplementary Tables

Table S1. Clinical perinatal characteristics group comparison, between VPT and FT, within a) RS-fMRI (VPT, n=31; FT, n=19) and b) dMRI (VPT, n=39; FT, n=18) datasets. Continuous variables were analysed using independent-samples t-tests and categorical variables using chi-squared tests. For each comparison, the test statistic with degrees of freedom (t(df)/χ^2(df), respectively), p-value, effect size (Cohen's d for continuous variables; Cramér's V for categorical variables), and 95% confidence intervals (95% CI [low, high]) are reported. Significant differences ($p<0.05$) are indicated in bold. VPT = very preterm infants. FT = full-term infants. Source data are provided as a Source Data file.

Clinical Characteristics	Data Sample	VPT vs FT<br>[t(df)/χ^2(df), p-value, d/Cramér's V, 95% CI]
GA at birth (weeks)	a) RS-fMRI	**t(48.0) = -20.364, p = < .001, d = -5.933, 95% CI [-11.141, -9.139]**
	b) dMRI	**t(55.0) = -19.128, p = < .001, d = -5.450, 95% CI [-11.448, -9.277]**
GA at TEA MRI scan (weeks)	a) RS-fMRI	t(48.0) = 0.399, p = 0.692, d = 0.116, 95% CI [-0.294, 0.440]
	b) dMRI	t(55.0) = -0.065, p = 0.949, d = -0.018, 95% CI [-0.370, 0.347]
Sex (Female), n (%)	a) RS-fMRI	χ^2(1) = 1.715, p = 0.190, Cramér's V = 0.185, 95% CI [-0.460, 0.049]
	b) dMRI	χ^2(1) = 0.000, p = 0.988, Cramér's V = 0.002, 95% CI [-0.292, 0.221]
SES	a) RS-fMRI	t(48.0) = 1.498, p = 0.141, d = 0.436, 95% CI [-0.479, 3.273]
	b) dMRI	t(55.0) = 1.541, p = 0.129, d = 0.439, 95% CI [-0.407, 3.117]
Birth weight (g)	a) RS-fMRI	**t(48.0) = -18.521, p = < .001, d = -5.396, 95% CI [-2211.198, -1778.123]**
	b) dMRI	**t(55.0) = -18.449, p = < .001, d = -5.257, 95% CI [-2290.026, -1841.256]**
Birth height (cm)	a) RS-fMRI	**t(48.0) = -13.234, p = < .001, d = -3.856, 95% CI [-13.337, -9.819]**
	b) dMRI	**t(55.0) = -11.394, p = < .001, d = -3.247, 95% CI [-14.249, -9.986]**
Head circumference at birth (cm)	a) RS-fMRI	**t(48.0) = -14.245, p = < .001, d = -4.150, 95% CI [-9.267, -6.975]**
	b) dMRI	**t(55.0) = -11.590, p = < .001, d = -3.303, 95% CI [-9.474, -6.680]**
APGAR at 1 min	a) RS-fMRI	**t(48.0) = -4.138, p = < .001, d = -1.206, 95% CI [-4.834, -1.672]**
	b) dMRI	**t(55.0) = -4.198, p = < .001, d = -1.196, 95% CI [-4.843, -1.713]**
APGAR at 5 min	a) RS-fMRI	**t(48.0) = -3.293, p = 0.002, d = -0.959, 95% CI [-2.283, -0.552]**
	b) dMRI	**t(55.0) = -3.379, p = 0.001, d = -0.963, 95% CI [-2.839, -0.725]**
IUGR, n (%)	a) RS-fMRI	χ^2(1) = 1.849, p = 0.174, Cramér's V = 0.192, 95% CI [-0.030, 0.326]
	b) dMRI	χ^2(1) = 1.677, p = 0.195, Cramér's V = 0.172, 95% CI [-0.040, 0.297]
BPD, n (%)	a) RS-fMRI	**χ^2(1) = 7.672, p = 0.006, Cramér's V = 0.392, 95% CI [0.162, 0.562]**
	b) dMRI	**χ^2(1) = 7.517, p = 0.006, Cramér's V = 0.363, 95% CI [0.162, 0.541]**
IVH (grade I), n (%)	a) RS-fMRI	χ^2(1) = 0.617, p = 0.432, Cramér's V = 0.111, 95% CI [-0.083, 0.249]
	b) dMRI	χ^2(1) = 0.326, p = 0.568, Cramér's V = 0.076, 95% CI [-0.106, 0.203]

Table S2. Clinical perinatal characteristics differences per group (VPT and FT) between MRI sequence samples, RS-fMRI (VPT, n=31; FT, n=19) vs dMRI (VPT, n=39; FT, n=18). Continuous variables were analysed using independent-samples t-tests and categorical variables using chi-squared tests. For each comparison, the test statistic with degrees of freedom (t(df)/χ^2(df), respectively), p-value, effect size (Cohen's d for continuous variables; Cramér's V for categorical variables), and 95% confidence intervals (95% CI [low, high]) are reported. VPT = very preterm infants. FT = full-term infants. Source data are provided as a Source Data file.

Clinical Characteristic	VPT (RS-fMRI vs dMRI data) [t(df)/χ^2(df), p-value, d/Cramér's V, 95% CI]	FT (RS-fMRI vs dMRI data) [t(df)/χ^2(df), p-value, d/Cramér's V, 95% CI]
GA at birth (weeks)	t(68.0) = 0.224, p = 0.824, d = 0.054, 95% CI [-0.890, 1.114]	t(35.0) = -0.304, p = 0.763, d = -0.100, 95% CI [-0.849, 0.628]
GA at 33w MRI scan (weeks)	t(68.0) = 0.553, p = 0.582, d = 0.133, 95% CI [-0.157, 0.277]	
GA at TEA MRI scan (weeks)	t(68.0) = -0.118, p = 0.907, d = -0.028, 95% CI [-0.275, 0.244]	t(35.0) = -0.395, p = 0.695, d = -0.130, 95% CI [-0.612, 0.412]
Sex (Female), n (%)	χ^2(1) = 0.072, p = 0.789, Cramér's V = 0.032	χ^2(1) = 0.218, p = 0.640, Cramér's V = 0.077
SES	t(68.0) = 0.162, p = 0.872, d = 0.039, 95% CI [-1.440, 1.695]	t(35.0) = 0.089, p = 0.929, d = 0.029, 95% CI [-1.841, 2.010]
Birth weight (g)	t(68.0) = 0.165, p = 0.869, d = 0.040, 95% CI [-167.935, 198.142]	t(35.0) = -0.442, p = 0.661, d = -0.145, 95% CI [-312.469, 200.715]
Birth height (cm)	t(68.0) = 0.401, p = 0.690, d = 0.096, 95% CI [-1.512, 2.272]	t(35.0) = -0.240, p = 0.812, d = -0.079, 95% CI [-1.510, 1.191]
Head circumference at birth (cm)	t(68.0) = -0.478, p = 0.634, d = -0.115, 95% CI [-1.543, 0.946]	t(35.0) = -0.611, p = 0.545, d = -0.201, 95% CI [-1.100, 0.591]
APGAR at 1 min	t(68.0) = 0.198, p = 0.843, d = 0.048, 95% CI [-1.364, 1.665]	t(35.0) = 0.244, p = 0.809, d = 0.080, 95% CI [-0.921, 1.172]
APGAR at 5 min	t(68.0) = 0.928, p = 0.357, d = 0.223, 95% CI [-0.510, 1.397]	t(35.0) = 0.283, p = 0.779, d = 0.093, 95% CI [-0.487, 0.645]
IUGR, n (%)	χ^2(1) = 0.000, p = 1.000, Cramér's V = 0.000	
BPD, n (%)	χ^2(1) = 0.000, p = 1.000, Cramér's V = 0.000	
IVH (grade I), n (%)	χ^2(1) = 0.000, p = 1.000, Cramér's V = 0.000	

Table S3. Longitudinal delta changes in BOLD variability (n=31) and cortical microstructure (n=39) in very preterm infants from 33 wGA to TEA (TEA – 33 wGA) per resting-state network. Metrics include: BOLD variability (BOLD SD), Mean Kurtosis (MK), Radial Kurtosis (RK), Axial Kurtosis (AK), Mean Diffusivity (MD), Fractional Anisotropy (FA), intra-neurite volume fraction (intra), intrinsic diffusivity (diff), extra-neurite mean diffusivity (extraMD), and extra-neurite transverse diffusivity (extraTrans). Two-sided paired t-tests were performed comparing 33 wGA to TEA within each RSN. Effect sizes are reported as Cohen's d. p-values were FDR-adjusted for multiple comparisons. The full inferential statistic is reported as: t(df) = value, p = value, d = value, 95% CI [low, high]. Values in bold indicate significant longitudinal changes that survived FDR correction (p < 0.05). TEA = term-equivalent age. Source data are provided as a Source Data file.

Cortical region	Metric	Mean delta (TEA – 33wGA)	SD	Statistic t(df) = value, p = value, effect size (d), 95% CI	p-value (FDR adj.)
**Precuneus**	BOLD SD	5.2775	7.5519	t(30) = 3.891, p = < .001, d = 0.699, [2.5074, 8.0475]	**0.001** **
	MK	-0.0366	0.0850	t(38) = -2.685, p = 0.011, d = -0.430, [-0.0641, -0.0090]	**0.032** *
	RK	-0.0550	0.0811	t(35) = -4.070, p = < .001, d = -0.678, [-0.0824, -0.0276]	**0.001** **
	AK	-0.0230	0.0581	t(37) = -2.440, p = 0.020, d = -0.396, [-0.0421, -0.0039]	0.111
	MD	-0.0003	0.0002	t(38) = -10.011, p = < .001, d = -1.603, [-0.0004, -0.0003]	**< .001** ***
	FA	-0.0196	0.0172	t(34) = -6.761, p = < .001, d = -1.143, [-0.0255, -0.0137]	**< .001** ***
	Intra	-0.0127	0.0382	t(37) = -2.045, p = 0.048, d = -0.332, [-0.0252, -0.0001]	0.054
	Diff	-0.0004	0.0003	t(37) = -7.529, p = < .001, d = -1.221, [-0.0005, -0.0003]	**< .001** ***
	ExtraMD	-0.0003	0.0002	t(37) = -8.862, p = < .001, d = -1.438, [-0.0004, -0.0002]	**< .001** ***
	ExtraTrans	-0.0003	0.0002	t(38) = -8.962, p = < .001, d = -1.435, [-0.0004, -0.0002]	**< .001** ***
**Posterior Cingulate Cortex**	BOLD SD	3.3285	4.0239	t(27) = 4.377, p = < .001, d = 0.827, [1.7682, 4.8888]	**< .001** ***
	MK	-0.0408	0.0628	t(37) = -4.003, p = < .001, d = -0.649, [-0.0614, -0.0201]	**0.003** **
	RK	-0.0555	0.0788	t(37) = -4.343, p = < .001, d = -0.705, [-0.0814, -0.0296]	**< .001** ***
	AK	-0.0165	0.0518	t(37) = -1.962, p = 0.057, d = -0.318, [-0.0335, 0.0005]	0.172
	MD	-0.0002	0.0001	t(37) = -11.160, p = < .001, d = -1.810, [-0.0002, -0.0002]	**< .001** ***
	FA	-0.0203	0.0120	t(35) = -10.120, p = < .001, d = -1.687, [-0.0244, -0.0162]	**< .001** ***
	Intra	-0.0164	0.0283	t(35) = -3.472, p = 0.001, d = -0.579, [-0.0260, -0.0068]	**0.002** **
	Diff	-0.0003	0.0002	t(35) = -8.198, p = < .001, d = -1.366, [-0.0004, -0.0002]	**< .001** ***
	ExtraMD	-0.0002	0.0002	t(36) = -8.303, p = < .001, d = -1.365, [-0.0003, -0.0002]	**< .001** ***
	ExtraTrans	-0.0002	0.0002	t(38) = -7.842, p = < .001, d = -1.256, [-0.0002, -0.0001]	**< .001** ***
**Sensorimotor**	BOLD SD	3.2260	3.8958	t(30) = 4.611, p = < .001, d = 0.828, [1.7970, 4.6550]	**< .001** ***
	MK	-0.0504	0.0854	t(36) = -3.588, p = < .001, d = -0.590, [-0.0789, -0.0219]	**0.004** **
	RK	-0.0540	0.0857	t(35) = -3.782, p = < .001, d = -0.630, [-0.0830, -0.0250]	**0.002** **
	AK	-0.0282	0.0731	t(36) = -2.343, p = 0.025, d = -0.385, [-0.0526, -0.0038]	0.111
	MD	-0.0001	0.0002	t(37) = -3.224, p = 0.003, d = -0.523, [-0.0001, -0.0000]	**0.003** **
	FA	-0.0171	0.0168	t(35) = -6.096, p = < .001, d = -1.016, [-0.0228, -0.0114]	**< .001** ***
	Intra	-0.0306	0.0521	t(35) = -3.528, p = 0.001, d = -0.588, [-0.0483, -0.0130]	**0.002** **
	Diff	-0.0002	0.0003	t(37) = -4.186, p = < .001, d = -0.679, [-0.0003, -0.0001]	**< .001** ***
	ExtraMD	-0.0001	0.0002	t(37) = -3.737, p = < .001, d = -0.606, [-0.0002, -0.0001]	**< .001** ***
	ExtraTrans	-0.0001	0.0002	t(38) = -2.657, p = 0.011, d = -0.425, [-0.0001, -0.0000]	**0.013** *
**Visual**	BOLD SD	3.0321	5.1561	t(26) = 3.056, p = 0.005, d = 0.588, [0.9924, 5.0718]	**0.009** **
	MK	-0.0256	0.0872	t(37) = -1.811, p = 0.078, d = -0.294, [-0.0543, 0.0030]	0.117
	RK	-0.0435	0.1086	t(37) = -2.468, p = 0.018, d = -0.400, [-0.0792, -0.0078]	**0.033** *
	AK	0.0001	0.0594	t(37) = 0.010, p = 0.992, d = 0.002, [-0.0194, 0.0196]	0.992
	MD	-0.0003	0.0002	t(36) = -9.492, p = < .001, d = -1.560, [-0.0003, -0.0002]	**< .001** ***
	FA	-0.0395	0.0209	t(35) = -11.348, p = < .001, d = -1.891, [-0.0465, -0.0324]	**< .001** ***
	Intra	-0.0352	0.0458	t(37) = -4.734, p = < .001, d = -0.768, [-0.0502, -0.0201]	**< .001** ***
	Diff	-0.0003	0.0002	t(35) = -8.792, p = < .001, d = -1.465, [-0.0004, -0.0003]	**< .001** ***
	ExtraMD	-0.0003	0.0002	t(34) = -9.066, p = < .001, d = -1.532, [-0.0003, -0.0002]	**< .001** ***

	ExtraTrans	-0.0002	0.0001	t(35) = -9.823, p = < .001, d = -1.637, [-0.0003, -0.0002]	**< .001** ***
**Auditory**	BOLD SD	2.5841	3.3268	t(30) = 4.325, p = < .001, d = 0.777, [1.3639, 3.8044]	**< .001** ***
	MK	-0.0307	0.0807	t(38) = -2.377, p = 0.023, d = -0.381, [-0.0569, -0.0046]	**0.041** *
	RK	-0.0314	0.0891	t(37) = -2.171, p = 0.036, d = -0.352, [-0.0607, -0.0021]	0.055
	AK	-0.0149	0.0540	t(37) = -1.705, p = 0.097, d = -0.277, [-0.0327, 0.0028]	0.217
	MD	-0.0001	0.0001	t(35) = -6.035, p = < .001, d = -1.006, [-0.0002, -0.0001]	**< .001** ***
	FA	-0.0276	0.0156	t(36) = -10.747, p = < .001, d = -1.767, [-0.0328, -0.0224]	**< .001** ***
	Intra	-0.0403	0.0305	t(35) = -7.927, p = < .001, d = -1.321, [-0.0506, -0.0300]	**< .001** ***
	Diff	-0.0002	0.0003	t(37) = -4.286, p = < .001, d = -0.695, [-0.0003, -0.0001]	**< .001** ***
	ExtraMD	-0.0001	0.0002	t(37) = -3.881, p = < .001, d = -0.630, [-0.0002, -0.0001]	**< .001** ***
	ExtraTrans	-0.0001	0.0002	t(38) = -3.315, p = 0.002, d = -0.531, [-0.0001, -0.0000]	**0.003** **
**Paralimbic**	BOLD SD	0.7642	4.8498	t(30) = 0.877, p = 0.387, d = 0.158, [-1.0147, 2.5431]	0.498
	MK	-0.0084	0.0658	t(38) = -0.801, p = 0.428, d = -0.128, [-0.0297, 0.0129]	0.551
	RK	-0.0231	0.0759	t(38) = -1.903, p = 0.065, d = -0.305, [-0.0477, 0.0015]	0.083
	AK	0.0072	0.0621	t(38) = 0.726, p = 0.472, d = 0.116, [-0.0129, 0.0274]	0.607
	MD	0.0000	0.0001	t(35) = -0.171, p = 0.865, d = -0.028, [-0.0000, 0.0000]	0.865
	FA	-0.0262	0.0127	t(34) = -12.231, p = < .001, d = -2.067, [-0.0305, -0.0218]	**< .001** ***
	Intra	-0.0696	0.0393	t(37) = -10.923, p = < .001, d = -1.772, [-0.0825, -0.0567]	**< .001** ***
	Diff	-0.0002	0.0002	t(36) = -7.501, p = < .001, d = -1.233, [-0.0003, -0.0002]	**< .001** ***
	ExtraMD	-0.0001	0.0001	t(36) = -5.509, p = < .001, d = -0.906, [-0.0002, -0.0001]	**< .001** ***
	ExtraTrans	-0.0001	0.0001	t(36) = -3.516, p = 0.001, d = -0.578, [-0.0001, -0.0000]	**0.002** **
**Thalamus**	BOLD SD	-0.3433	5.5546	t(28) = -0.333, p = 0.742, d = -0.062, [-2.4562, 1.7695]	0.742
	MK	0.0075	0.0864	t(37) = 0.533, p = 0.597, d = 0.087, [-0.0209, 0.0359]	0.672
	RK	0.0022	0.1019	t(37) = 0.136, p = 0.893, d = 0.022, [-0.0312, 0.0357]	0.893
	AK	0.0104	0.0735	t(38) = 0.885, p = 0.382, d = 0.142, [-0.0134, 0.0342]	0.573
	MD	-0.0002	0.0001	t(37) = -12.239, p = < .001, d = -1.985, [-0.0002, -0.0001]	**< .001** ***
	FA	0.0162	0.0189	t(37) = 5.298, p = < .001, d = 0.859, [0.0100, 0.0224]	**< .001** ***
	Intra	0.0103	0.0356	t(37) = 1.784, p = 0.083, d = 0.289, [-0.0014, 0.0220]	0.083
	Diff	-0.0001	0.0002	t(37) = -4.003, p = < .001, d = -0.649, [-0.0002, -0.0001]	**< .001** ***
	ExtraMD	-0.0001	0.0001	t(37) = -6.185, p = < .001, d = -1.003, [-0.0002, -0.0001]	**< .001** ***
	ExtraTrans	-0.0002	0.0001	t(37) = -8.041, p = < .001, d = -1.304, [-0.0002, -0.0001]	**< .001** ***
**Limbic**	BOLD SD	-0.4090	4.8056	t(27) = -0.450, p = 0.656, d = -0.085, [-2.2724, 1.4545]	0.738
	MK	-0.0262	0.0631	t(37) = -2.554, p = 0.015, d = -0.414, [-0.0469, -0.0054]	**0.034** *
	RK	-0.0377	0.0728	t(37) = -3.192, p = 0.003, d = -0.518, [-0.0616, -0.0138]	**0.006** **
	AK	-0.0085	0.0567	t(38) = -0.938, p = 0.354, d = -0.150, [-0.0269, 0.0099]	0.573
	MD	0.0000	0.0001	t(37) = -1.374, p = 0.178, d = -0.223, [-0.0001, 0.0000]	0.200
	FA	-0.0098	0.0126	t(35) = -4.673, p = < .001, d = -0.779, [-0.0141, -0.0056]	**< .001** ***
	Intra	-0.0307	0.0343	t(36) = -5.435, p = < .001, d = -0.893, [-0.0421, -0.0192]	**< .001** ***
	Diff	-0.0002	0.0002	t(34) = -9.168, p = < .001, d = -1.550, [-0.0003, -0.0002]	**< .001** ***
	ExtraMD	-0.0002	0.0001	t(33) = -9.055, p = < .001, d = -1.553, [-0.0002, -0.0001]	**< .001** ***
	ExtraTrans	-0.0001	0.0001	t(35) = -6.778, p = < .001, d = -1.130, [-0.0002, -0.0001]	**< .001** ***
**Prefrontal Cortex**	BOLD SD	-2.0539	10.152	t(24) = -1.012, p = 0.322, d = -0.202, [-6.2443, 2.1365]	0.483
	MK	-0.0058	0.0878	t(37) = -0.410, p = 0.684, d = -0.066, [-0.0347, 0.0230]	0.684
	RK	-0.0193	0.1022	t(37) = -1.164, p = 0.252, d = -0.189, [-0.0529, 0.0143]	0.283
	AK	0.0068	0.0752	t(37) = 0.560, p = 0.579, d = 0.091, [-0.0179, 0.0316]	0.651
	MD	0.0001	0.0002	t(38) = 4.422, p = < .001, d = 0.708, [0.0001, 0.0002]	**< .001** ***
	FA	-0.0432	0.0195	t(37) = -13.634, p = < .001, d = -2.212, [-0.0496, -0.0368]	**< .001** ***
	Intra	-0.0615	0.0495	t(38) = -7.753, p = < .001, d = -1.242, [-0.0776, -0.0454]	**< .001** ***
	Diff	0.0000	0.0002	t(36) = -0.750, p = 0.458, d = -0.123, [-0.0001, 0.0000]	0.458
	ExtraMD	0.0000	0.0002	t(37) = 0.846, p = 0.403, d = 0.137, [-0.0000, 0.0001]	0.403
	ExtraTrans	0.0000	0.0002	t(37) = 1.327, p = 0.193, d = 0.215, [-0.0000, 0.0001]	0.193

Table S4. Gene Ontology enrichment of genes that increased significantly more in group 1 than group 2, from mid- to late-fetal period, using background reference set (5,287 genes). Gene Ontology enrichment analysis was performed using WebGestalt, Fisher's exact test, FDR<0.05.

		Reference set		
		Fetal gene markers (n=5287)		
GO term†	Description	Enrichment	FDR	Genes
GO:0007272	ensheathment of neurons	6.15	0.00001*	CD9; CLDN11; COL6A1; ERBB3; MAG; MAL; MBP; MOBP; NKX6-2; PLLP; PLP1; PMP22; TNFRSF1B; UGT8
GO:0042063	gliogenesis	2.85	0.01796*	APCDD1; CD9; COL6A1; ERBB3; GPR17; GPR183; GPR37L1; IL6ST; MAG; MAL; MOBP; NKX6-2; PLP1; TNFRSF1B
GO:0043062	extracellular structure organization	3.58	0.01796*	CAV1; CAV2; COL18A1; COL5A2; COL6A1; EFEMP2; LAMC1; SLC39A8; TNFRSF1B; VWA1
GO:0045229	external encapsulating structure organization	3.58	0.01796*	CAV1; CAV2; COL18A1; COL5A2; COL6A1; EFEMP2; LAMC1; SLC39A8; TNFRSF1B; VWA1
GO:0019221	cytokine-mediated signaling pathway	3.61	0.00797*	CAV1; CD74; F3; GPR17; IFI27; IL6ST; IRF1; MX1; OAS2; PADI2; PTPRC; SP100; TNFRSF1B
GO:0050817	coagulation	4.10	0.01351*	ACTN1; CAV1; CD9; CSRP1; F3; IL6ST; MMRN1; NFE2L2; PAPSS2; STXBP3
GO:0042060	wound healing	3.04	0.01365*	ACTN1; CAV1; CD9; CSRP1; ELK3; ERBB3; F3; GATA2; HBEGF; IL6ST; MMRN1; NFE2L2; PAPSS2; STXBP3
GO:0097191	extrinsic apoptotic signaling pathway	3.84	0.01548*	BAG3; CAV1; ERBB3; IFI27; MAL; PTPRC; SH3RF1; SP100; STK3; TNFRSF1B
GO:0033002	muscle cell proliferation	3.86	0.01796*	APOD; CALCRL; CAV2; EFEMP2; HBEGF; IGFBP5; JUN; NDRG2; PTGS2
GO:0050878	regulation of body fluid levels	3.55	0.00797*	ACTN1; CAV1; CD9; CSRP1; EMP2; F3; IL6ST; MMRN1; NFE2L2; OAS2; PAPSS2; STXBP3; WFS1

*p<0.05 after correction for multiple comparisons using FDR

† top 10 terms with 'fetal gene markers' background set are listed

Table S5. Effect of preterm birth on BOLD variability and cortical microstructure at TEA per region. Metrics include: BOLD variability (BOLD SD), Mean Kurtosis (MK), Radial Kurtosis (RK), Axial Kurtosis (AK), Mean Diffusivity (MD), Extra-neurite Mean Diffusivity (ExtraMD), Extra-neurite Transverse Diffusivity (ExtraTrans), intrinsic diffusivity (Diff). Two-sided independent t-tests were performed comparing FT and VPT groups within each RSN, and p-values were FDR-adjusted for multiple comparisons. Numbers in bold indicate significant results between groups that survived FDR correction ($p \leq 0.05$). VPT = very preterm infants. FT = full-term infants. TEA = term-equivalent age. Source data are provided as a Source Data file.

Cortical region	Metric	Mean measurement		Mean Difference (VPT-FT) (95% CI)	Group-effect
		VPT	FT		p-value (FDR adjusted)
**Precuneus**	BOLD SD	15.9	17.6	-1.71 (-4.9, 1.5)	0.33
	MK	0.42	0.38	0.04 (0.0002, 0.007)	0.06
	RK	0.41	0.34	0.06 (0.02, 0.12)	**0.012***
	AK	0.453	0.456	-0.003 (-0.02, 0.02)	0.89
	MD	0.0017	0.0014	0.0003 (0.00022, 0.00036)	< **0.001*****
	ExtraMD	0.0018	0.0015	0.0003 (0.0002, 0.0004)	< **0.001*****
	ExtraTrans	0.0017	0.0014	0.0003 (0.0002, 0.0004)	< **0.001*****
	Diff	0.0020	0.0017	0.0003 (0.0002, 0.0004)	< **0.001*****
**Posterior Cingulate Cortex**	BOLD SD	14.4	18.6	-4.21 (-7.8, -0.6)	**0.04***
	MK	0.43	0.39	0.03 (-0.003, 0.065)	0.07
	RK	0.39	0.33	0.06 (0.02, 0.11)	**0.011***
	AK	0.48	0.48	0.0001 (0.02, 0.02)	0.99
	MD	0.0014	0.0013	0.0001 (0.00006, 0.0001)	< **0.001*****
	ExtraMD	0.0015	0.0014	0.0001 (0.00007, 0.0002)	< **0.001*****
	ExtraTrans	0.0015	0.0014	0.0001 (0.00008, 0.0002)	< **0.001*****
	Diff	0.0017	0.0016	0.0001 (0.00007, 0.0002)	< **0.001*****
**Sensorimotor**	BOLD SD	10.0	12.9	-2.94 (-5.8, 0.01)	0.007
	MK	0.47	0.43	0.04 (0.003, 0.076)	**0.049***
	RK	0.44	0.37	0.07 (0.02, 0.12)	**0.011***
	AK	0.52	0.53	-0.01 (-0.04, 0.015)	0.58
	MD	0.0017	0.0013	0.0004 (0.0003, 0.0005)	< **0.001*****
	ExtraMD	0.0019	0.0015	0.0004 (0.0003, 0.0005)	< **0.001*****
	ExtraTrans	0.0018	0.0014	0.0004 (0.0003, 0.0005)	< **0.001*****
	Diff	0.0020	0.0017	0.0003 (0.0002, 0.0004)	< **0.001*****
**Visual**	BOLD SD	16.2	23.1	-6.84 (-11.3, -2.3)	**0.012***
	MK	0.42	0.36	0.05 (0.01, 0.09)	**0.029***
	RK	0.38	0.30	0.08 (0.02, 0.13)	**0.011***
	AK	0.47	0.46	0.01 (-0.01, 0.03)	0.67
	MD	0.0014	0.0013	0.0001 (0.00006, 0.0002)	**0.0003*****
	ExtraMD	0.0016	0.0015	0.0001 (0.00004, 0.0002)	**0.007****
	ExtraTrans	0.0015	0.0014	0.0001 (0.00006, 0.0002)	**0.001****
	Diff	0.0018	0.0017	0.0001 (0.00012, 0.0002)	**0.03***
**Auditory**	BOLD SD	10.4	14.8	-4.42 (-7.1, -1.6)	**0.012***
	MK	0.46	0.40	0.06 (0.02, 0.10)	**0.024***
	RK	0.44	0.35	0.09 (0.02, 0.15)	**0.011***
	AK	0.50	0.48	0.01 (-0.009, 0.03)	0.58
	MD	0.0016	0.0014	0.00019 (0.00013, 0.00024)	< **0.001*****
	ExtraMD	0.0018	0.0015	0.00026 (0.00017, 0.00034)	< **0.001*****
	ExtraTrans	0.0016	0.0014	0.00023 (0.00016, 0.0003)	< **0.001*****
	Diff	0.0020	0.0017	0.0003 (0.0002, 0.0004)	< **0.001*****
**Paralimbic**	BOLD SD	11.4	13.2	-1.77 (-4.6, 1.1)	0.28

	MK	0.46	0.40	0.05 (0.02, 0.08)	**0.024***
	RK	0.43	0.36	0.07 (0.02, 0.11)	**0.011***
	AK	0.49	0.48	0.01(-0.01, 0.04)	0.58
	MD	0.0017	0.0014	0.00028 (0.0001, 0.0003)	< **0.001*****
	ExtraMD	0.0018	0.0016	0.0002 (0.0001, 0.0003)	< **0.001*****
	ExtraTrans	0.0017	0.0015	0.0002 (0.0001, 0.0003)	< **0.001*****
	Diff	0.0020	0.0018	0.0002 (0.0001, 0.0003)	< **0.001*****
**Thalamus**	BOLD SD	10.7	19.5	-8.73 (-11.9, -5.5)	< **0.001*****
	MK	0.38	0.35	0.026 (-0.01, 0.06)	0.17
	RK	0.33	0.29	0.04 (-0.006, 0.09)	0.08
	AK	0.43	0.42	0.01 (-0.01, 0.05)	0.58
	MD	0.0012	0.0011	0.0001 (0.00003, 0.0001)	**0.0006**
	ExtraMD	0.0013	0.0012	0.0001 (0.00005, 0.0001)	< **0.001*****
	ExtraTrans	0.0012	0.0011	0.0001 (0.00004, 0.0001)	< **0.001*****
	Diff	0.0015	0.0013	0.0002 (0.00008, 0.00025)	< **0.001*****
**Limbic**	BOLD SD	10.0	13.5	-3.45 (-6.3, -0.5)	**0.04***
	MK	0.44	0.40	0.04 (0.01, 0.08)	**0.024***
	RK	0.40	0.34	0.06 (0.01, 0.10)	**0.011***
	AK	0.50	0.48	0.01 (-0.01, 0.04)	0.58
	MD	0.0015	0.0013	0.0002 (0.00014, 0.00025)	< **0.001*****
	ExtraMD	0.0017	0.0015	0.0002 (0.00012, 0.00026)	< **0.001*****
	ExtraTrans	0.0016	0.0014	0.0002 (0.00014, 0.00026)	< **0.001*****
	Diff	0.0020	0.0018	0.0002 (0.00012, 0.00032)	< **0.001*****
**Prefrontal Cortex**	BOLD SD	17.6	19.8	-2.14 (-7.4, 3.1)	0.41
	MK	0.47	0.40	0.06 (0.01, 0.12)	**0.029***
	RK	0.44	0.34	0.10 (0.03, 0.16)	**0.011***
	AK	0.51	0.50	0.01 (-0.02, 0.05)	0.63
	MD	0.0018	0.0014	0.0004 (0.0003, 0.0005)	< **0.001*****
	ExtraMD	0.0020	0.0015	0.0004 (0.0003, 0.0005)	< **0.001*****
	ExtraTrans	0.0018	0.0014	0.0004 (0.0003, 0.0005)	< **0.001*****
	Diff	0.0022	0.0017	0.0005 (0.0004, 0.0006)	< **0.001*****
