# Supplementary material for: Regional BOLD variability reflects microstructural maturation and neuronal ensheathment in the preterm infant cortex
Source: Nat Commun. 2026 Apr 9;17:4849. doi: 10.1038/s41467-026-71415-x (PMC13222875; doi:10.1038/s41467-026-71415-x)
Supplement: Supplementary file 2 — Reporting Summary [file 41467_2026_71415_MOESM2_ESM.pdf]

nature portfolio

NCOMMS-25-60502
Corresponding author(s): Joana Sa de Almeida
Last updated by author(s): 15.01.2025

Reporting Summary

Nature Portfolio wishes to improve the reproducibility of the work that we publish. This form provides structure for consistency and transparency in reporting. For further information on Nature Portfolio policies, see our Editorial Policies and the Editorial Policy Checklist.

Statistics

For all statistical analyses, confirm that the following items are present in the figure legend, table legend, main text, or Methods section.

n/a	Confirmed	
☐	☒	The exact sample size (*n*) for each experimental group/condition, given as a discrete number and unit of measurement
☐	☒	A statement on whether measurements were taken from distinct samples or whether the same sample was measured repeatedly
☐	☒	The statistical test(s) used AND whether they are one- or two-sided<br>*Only common tests should be described solely by name; describe more complex techniques in the Methods section.*
☐	☒	A description of all covariates tested
☐	☒	A description of any assumptions or corrections, such as tests of normality and adjustment for multiple comparisons
☐	☒	A full description of the statistical parameters including central tendency (e.g. means) or other basic estimates (e.g. regression coefficient) AND variation (e.g. standard deviation) or associated estimates of uncertainty (e.g. confidence intervals)
☐	☒	For null hypothesis testing, the test statistic (e.g. *F*, *t*, *r*) with confidence intervals, effect sizes, degrees of freedom and *P* value noted<br>*Give P values as exact values whenever suitable.*
☒	☐	For Bayesian analysis, information on the choice of priors and Markov chain Monte Carlo settings
☒	☐	For hierarchical and complex designs, identification of the appropriate level for tests and full reporting of outcomes
☒	☐	Estimates of effect sizes (e.g. Cohen's *d*, Pearson's *r*), indicating how they were calculated

Our web collection on statistics for biologists contains articles on many of the points above.

Software and code

Policy information about availability of computer code

Data collection	No specific software or code used for MRI data or genetic data collection.
Data analysis	Software and code used in this study for MRI analysis are publicly available as part of FSL v5.0.10 (https://fsl.fmrib.ox.ac.uk/fsl/fslwiki/), MRtrix3 (Tournier et al., 2019), SMT (https://github.com/ekaden/smt) and DIPY (https://docs.dipy.org/stable/interfaces/reconstruction_flow.html) software packages. dMRI data were pre-processed using EDDY command adapted for neonatal motion, from the neonatal dMRI automated pipeline from developing Human Connectome Project (dHCP, http://www.developingconnectome.org), and can be found at: https://git.fmrib.ox.ac.uk/matteob/dHCP_neo_dMRI_pipeline_release (Bastiani et al., 2019). Developmental RNA-seq data used in this study were downloaded from: http://development.psychencode.org and have been processed using RSEQtools (version 0.5). All statistic analysis were performed using R (4.4.1) software, apart from gene set enrichment analysis (GSEA),which was performed using WebGestalt (Wang et al., 2017). Supporting code for this manuscript, used to generate the results and figures is available on Zenodo (DOI: https://doi.org/10.5281/zenodo.18875986).

For manuscripts utilizing custom algorithms or software that are central to the research but not yet described in published literature, software must be made available to editors and reviewers. We strongly encourage code deposition in a community repository (e.g. GitHub). See the Nature Portfolio guidelines for submitting code & software for further information.

Data

Policy information about availability of data

All manuscripts must include a data availability statement. This statement should provide the following information, where applicable:

- Accession codes, unique identifiers, or web links for publicly available datasets
- A description of any restrictions on data availability
- For clinical datasets or third party data, please ensure that the statement adheres to our policy

All neuroimaging data were acquired in the context of a research project approved by the ethical committee in 2016. The raw data are protected and are not available due to data privacy laws. Developmental RNA-seq data used in this study were downloaded from: http://development.psychencode.org/. Source Data are provided with this paper.

Research involving human participants, their data, or biological material

Policy information about studies with human participants or human data. See also policy information about sex, gender (identity/presentation), and sexual orientation and race, ethnicity and racism.

Reporting on sex and gender	We have always described the sex attributed at birth (biological attribute). Given the sample size, the study was not sufficiently powered to support meaningful sex-stratified analyses, reason why no post hoc sex-based analyses were conducted. Regarding the MRI data, we have reported the differences in sex between our groups, which were not significant across the different MRI modalities. For the genetic data, obtained from the open source database, sex is also reported per specimen ID, and used as a fixed factor when modeling significant changes in gene expression as a function of regional cluster (1 and 2) and time using a Linear mixed effects regression model (LMM). Sex-based analyses were not a primary objective of this study and were therefore not performed a priori.
Reporting on race, ethnicity, or other socially relevant groupings	We have reported the socio-economic score (SES) per group in our clinical MRI data set. SES was calculated according to Largo et al. 1989 definition, and was not significantly different between groups across the different MRI modalities.
Population characteristics	54 very preterm infants (VPT, born <32 weeks Gestation Age, GA) and 24 full-term (FT) born infants were recruited at birth. Out study comprises both longitudinal and cross-sectional designs. The longitudinal design involves only our VPT infant cohort, who underwent MRI examinations at two time-points, firstly during the 33rd week of GA, and secondly at term-equivalent age (TEA). The cross-sectional design involves both the VPT at TEA and the FT newborns, who underwent a single MRI examination few days after being born (term age).<br>Significant differences between the VPT and FT groups were observed, as expected, in the following perinatal variables: GA at birth, birth weight, birth height, birth head circumference, APGAR at 1 and 5 minutes and incidence of bronchopulmonary dysplasia (BPD). There were no differences between groups in sex, GA at 2nd MRI scan (TEA), neonatal asphyxia, intrauterine growth restriction, intraventricular hemorrhage grade I, and socio-economic parental status scores.
Recruitment	Patient were recruited at the neonatal and maternity units of the University Hospitals of Geneva (HUG), Switzerland, from 2017 to 2020. Exclusion criteria for all babies included major brain lesions detected on the MRI, such as high-grade intraventricular hemorrhage, leukomalacia, as well as micro or macrocephaly or congenital syndromes.
Ethics oversight	Research Ethics Committee approval was granted by the “Cantonal Research Ethics Committee of Geneva”, and hosted at University Hospitals of Geneva (HUG). Written parental consent was obtained prior to infant’s participation to the study.

Note that full information on the approval of the study protocol must also be provided in the manuscript.

Field-specific reporting

Please select the one below that is the best fit for your research. If you are not sure, read the appropriate sections before making your selection.

[x] Life sciences   [ ] Behavioural & social sciences   [ ] Ecological, evolutionary & environmental sciences

For a reference copy of the document with all sections, see nature.com/documents/nr-reporting-summary-flat.pdf

Life sciences study design

All studies must disclose on these points even when the disclosure is negative.

Sample size	Based on previous studies in similar cohorts and accounting for potential dropouts, we initially anticipated recruiting approximately 60 very preterm (VPT) infants and 30 full-term (FT) newborns during the study period (2017–2020). This sample would provide ~86% statistical power to detect a large effect size of 0.8 (i.e., a difference of 0.8 standard deviations in BOLD or diffusion metrics between VPT and FT infants at term-equivalent age). For a moderate effect size of 0.5, the power would decrease to ~48%. The final sample included 54 VPT infants and 24 FT newborns. Sample size was primarily determined by parental acceptance during recruitment. Prior studies in similar populations suggested that this sample size could provide sufficient power to detect meaningful longitudinal changes in VPT infants and cross-sectional differences at term-equivalent age (TEA) between VPT and FT infants in BOLD and diffusion MRI analysis.
Data exclusions	Exclusion criteria for all babies included major brain lesions detected on the MRI, such as high-grade intraventricular hemorrhage, leukomalacia, as well as micro or macrocephaly or congenital syndromes. Infants whose MRI protocol acquisition was incomplete (not comprising a T2-weighted image, resting-state fMRI (RS-fMRI) sequence and multi-shell diffusion imaging (MSDI) sequence), without both

longitudinal time-points (in preterm infants' case) or whose images presented excessive motion were excluded from the analysis.

Replication	Not the aim of the study and thus not performed.
Randomization	There was no need for randomization in experimental groups. The groups used in the MRI data analysis were established based on the Gestational Age (GA) at birth. Very preterm infants (VPT) are those born <32 weeks GA, and Full-Term (FT) infants are those born after 37 weeks GA.
Blinding	Blinding was not applicable because group allocation was based on gestational age at birth (very preterm vs. full-term), which is inherent and known at recruitment. Knowing the group was essential, as the study's purpose is to compare these populations.

Reporting for specific materials, systems and methods

We require information from authors about some types of materials, experimental systems and methods used in many studies. Here, indicate whether each material, system or method listed is relevant to your study. If you are not sure if a list item applies to your research, read the appropriate section before selecting a response.

Materials & experimental systems

n/a	Involved in the study	
☐	☐	Antibodies
☐	☐	Eukaryotic cell lines
☐	☐	Palaeontology and archaeology
☐	☐	Animals and other organisms
☐	☒	Clinical data
☐	☐	Dual use research of concern
☐	☐	Plants

Methods

n/a	Involved in the study	
☐	☐	ChIP-seq
☐	☐	Flow cytometry
☐	☒	MRI-based neuroimaging

Antibodies

Antibodies used	*Describe all antibodies used in the study; as applicable, provide supplier name, catalog number, clone name, and lot number.*
Validation	*Describe the validation of each primary antibody for the species and application, noting any validation statements on the manufacturer's website, relevant citations, antibody profiles in online databases, or data provided in the manuscript.*

Eukaryotic cell lines

Policy information about cell lines and Sex and Gender in Research

Cell line source(s)	*State the source of each cell line used and the sex of all primary cell lines and cells derived from human participants or vertebrate models.*
Authentication	*Describe the authentication procedures for each cell line used OR declare that none of the cell lines used were authenticated.*
Mycoplasma contamination	*Confirm that all cell lines tested negative for mycoplasma contamination OR describe the results of the testing for mycoplasma contamination OR declare that the cell lines were not tested for mycoplasma contamination.*
Commonly misidentified lines (See ICLAC register)	*Name any commonly misidentified cell lines used in the study and provide a rationale for their use.*

Palaeontology and Archaeology

Specimen provenance	*Provide provenance information for specimens and describe permits that were obtained for the work (including the name of the issuing authority, the date of issue, and any identifying information). Permits should encompass collection and, where applicable, export.*
Specimen deposition	*Indicate where the specimens have been deposited to permit free access by other researchers.*
Dating methods	*If new dates are provided, describe how they were obtained (e.g. collection, storage, sample pretreatment and measurement), where they were obtained (i.e. lab name), the calibration program and the protocol for quality assurance OR state that no new dates are provided.*

☐ Tick this box to confirm that the raw and calibrated dates are available in the paper or in Supplementary Information.

Ethics oversight	*Identify the organization(s) that approved or provided guidance on the study protocol, OR state that no ethical approval or guidance was required and explain why not.*

Note that full information on the approval of the study protocol must also be provided in the manuscript.

Animals and other research organisms

Policy information about studies involving animals; ARRIVE guidelines recommended for reporting animal research, and Sex and Gender in Research

Laboratory animals	*For laboratory animals, report species, strain and age OR state that the study did not involve laboratory animals.*
Wild animals	*Provide details on animals observed in or captured in the field; report species and age where possible. Describe how animals were caught and transported and what happened to captive animals after the study (if killed, explain why and describe method; if released, say where and when) OR state that the study did not involve wild animals.*
Reporting on sex	*Indicate if findings apply to only one sex; describe whether sex was considered in study design, methods used for assigning sex. Provide data disaggregated for sex where this information has been collected in the source data as appropriate; provide overall numbers in this Reporting Summary. Please state if this information has not been collected. Report sex-based analyses where performed, justify reasons for lack of sex-based analysis.*
Field-collected samples	*For laboratory work with field-collected samples, describe all relevant parameters such as housing, maintenance, temperature, photoperiod and end-of-experiment protocol OR state that the study did not involve samples collected from the field.*
Ethics oversight	*Identify the organization(s) that approved or provided guidance on the study protocol, OR state that no ethical approval or guidance was required and explain why not.*

Note that full information on the approval of the study protocol must also be provided in the manuscript.

Clinical data

Policy information about clinical studies

All manuscripts should comply with the ICMJE guidelines for publication of clinical research and a completed CONSORT checklist must be included with all submissions.

Clinical trial registration	This is an observational study and not a clinical trial, comprises both longitudinal and cross-sectional designs.
Study protocol	Study protocol is described in the manuscript but not elsewhere.
Data collection	Infants that have undergone the MRI were recruited at the neonatal and maternity units of the University Hospitals of Geneva (HUG), Switzerland, from 2017 to 2020. Parents who agree to participate may differ from those who decline (e.g., more health-conscious, higher SES, or more motivated). This could affect generalizability. In addition, since the recruitment was done in one single hospital, the sample may not represent the broader population of preterm or full-term infants.
Outcomes	In this study, we aimed to investigate the maturation of BOLD variability in very preterm (VPT) infants, longitudinally, from 33 to 40-weeks gestational age (GA), across different resting-state networks (RSNs). In addition, using a comprehensive dMRI analysis, combining metrics from DTI, DKI, and SMT models, we evaluated how observed changes in BOLD variability across the cortex align with regional measures of cortical microstructure.<br>Furthermore, to better understand the biological correlates of the observed functional and microstructural changes, we examined spatiotemporal patterns of gene expression, in postmortem tissue samples over the same time period.<br>Finally, we aimed to assess weather preterm birth impacts the expected developmental maturation of BOLD variability and cortical microstructure, in comparison to full-term (FT) birth.

Dual use research of concern

Policy information about dual use research of concern

Hazards

Could the accidental, deliberate or reckless misuse of agents or technologies generated in the work, or the application of information presented in the manuscript, pose a threat to:

No	Yes	
☒	☐	Public health
☒	☐	National security
☒	☐	Crops and/or livestock
☒	☐	Ecosystems
☒	☐	Any other significant area

Experiments of concern

Does the work involve any of these experiments of concern:

No	Yes	
☒	☐	Demonstrate how to render a vaccine ineffective
☒	☐	Confer resistance to therapeutically useful antibiotics or antiviral agents
☒	☐	Enhance the virulence of a pathogen or render a nonpathogen virulent
☒	☐	Increase transmissibility of a pathogen
☒	☐	Alter the host range of a pathogen
☒	☐	Enable evasion of diagnostic/detection modalities
☒	☐	Enable the weaponization of a biological agent or toxin
☒	☐	Any other potentially harmful combination of experiments and agents

Plants

Seed stocks	*Report on the source of all seed stocks or other plant material used. If applicable, state the seed stock centre and catalogue number. If plant specimens were collected from the field, describe the collection location, date and sampling procedures.*
Novel plant genotypes	*Describe the methods by which all novel plant genotypes were produced. This includes those generated by transgenic approaches, gene editing, chemical/radiation-based mutagenesis and hybridization. For transgenic lines, describe the transformation method, the number of independent lines analyzed and the generation upon which experiments were performed. For gene-edited lines, describe the editor used, the endogenous sequence targeted for editing, the targeting guide RNA sequence (if applicable) and how the editor was applied.*
Authentication	*Describe any authentication procedures for each seed stock used or novel genotype generated. Describe any experiments used to assess the effect of a mutation and, where applicable, how potential secondary effects (e.g. second site T-DNA insertions, mosiacism, off-target gene editing) were examined.*

ChIP-seq

Data deposition

☐ Confirm that both raw and final processed data have been deposited in a public database such as GEO.

☐ Confirm that you have deposited or provided access to graph files (e.g. BED files) for the called peaks.

Data access links<br>*May remain private before publication.*	*For "Initial submission" or "Revised version" documents, provide reviewer access links. For your "Final submission" document, provide a link to the deposited data.*
Files in database submission	*Provide a list of all files available in the database submission.*
Genome browser session<br>(e.g. UCSC)	*Provide a link to an anonymized genome browser session for "Initial submission" and "Revised version" documents only, to enable peer review. Write "no longer applicable" for "Final submission" documents.*

Methodology

Replicates	*Describe the experimental replicates, specifying number, type and replicate agreement.*
Sequencing depth	*Describe the sequencing depth for each experiment, providing the total number of reads, uniquely mapped reads, length of reads and whether they were paired- or single-end.*
Antibodies	*Describe the antibodies used for the ChIP-seq experiments; as applicable, provide supplier name, catalog number, clone name, and lot number.*
Peak calling parameters	*Specify the command line program and parameters used for read mapping and peak calling, including the ChIP, control and index files used.*
Data quality	*Describe the methods used to ensure data quality in full detail, including how many peaks are at FDR 5% and above 5-fold enrichment.*
Software	*Describe the software used to collect and analyze the ChIP-seq data. For custom code that has been deposited into a community repository, provide accession details.*

Flow Cytometry

Plots

Confirm that:

- [ ] The axis labels state the marker and fluorochrome used (e.g. CD4-FITC).
- [ ] The axis scales are clearly visible. Include numbers along axes only for bottom left plot of group (a 'group' is an analysis of identical markers).
- [ ] All plots are contour plots with outliers or pseudocolor plots.
- [ ] A numerical value for number of cells or percentage (with statistics) is provided.

Methodology

Sample preparation	*Describe the sample preparation, detailing the biological source of the cells and any tissue processing steps used.*
Instrument	*Identify the instrument used for data collection, specifying make and model number.*
Software	*Describe the software used to collect and analyze the flow cytometry data. For custom code that has been deposited into a community repository, provide accession details.*
Cell population abundance	*Describe the abundance of the relevant cell populations within post-sort fractions, providing details on the purity of the samples and how it was determined.*
Gating strategy	*Describe the gating strategy used for all relevant experiments, specifying the preliminary FSC/SSC gates of the starting cell population, indicating where boundaries between "positive" and "negative" staining cell populations are defined.*

- [ ] Tick this box to confirm that a figure exemplifying the gating strategy is provided in the Supplementary Information.

Magnetic resonance imaging

Experimental design

Design type	fMRI resting-state
Design specifications	No experimental design applicable.
Behavioral performance measures	Not applicable

Acquisition

Imaging type(s)	structural T2-weighted image, fMRI resting-state and Multi-shell Diffusion MRI
Field strength	3.0T
Sequence & imaging parameters	MRI acquisition at both time-points was performed on a 3.0T Siemens Magnetom MR scanner (Siemens, Erlangen, Germany), using a 16-channel neonatal head coil.<br>T2-weighted images were acquired using the following parameters: 113 coronal slices, TR=4990 ms, TE=160 ms, flip angle=150°, matrix size=256×164; voxel size=0.8×0.8×1.2 mm3.<br>RS-fMRI data acquisition was obtained by means of T2*-weighted gradient echo echo-planar imaging (EPI) sequence with the following parameters: 590 images, TR = 700 ms, TE = 30 ms, 36 slices, voxel size = 2.5 x 2.5 x 2.5 mm3, flip angle = 60°, multiband factor = 4.
Area of acquisition	Brain MRI acquisition

Diffusion MRI [x] Used [ ] Not used

Parameters: Multi-shell diffusion imaging (MSDI) was acquired with a single-shot spin echo echo-planar imaging (SE-EPI) Stejksal-Tanner sequence with the following parameters TE=85 ms, TR=3170 ms, voxel size 1.8×1.8×1.8 mm3, multi-band acceleration factor of 2, GRAPPA 2. Images were acquired in the axial plane, in anterior-posterior (AP) phase encoding (PE) direction, with 4 volumes without diffusion-weighting (b0); 10 non-collinear directions with b=200 s/mm2, 30 non-collinear directions with b=1000 s/mm2; 50 non-collinear directions with b=2000 s/mm2. Additional b0 images were collected with reversed phase-encode blips, AP and posterior-anterior (PA), resulting in pairs of images with distortions going in opposite directions.

Preprocessing

Preprocessing software	Software and code used in this study are publicly available as part of FSL v5.0.10 (https://fsl.fmrib.ox.ac.uk/fsl/fslwiki/), MRtrix3 (Tournier et al., 2019), SMT (https://github.com/ekaden/smt), DIPY (https://docs.dipy.org/stable/interfaces/reconstruction_flow.html), SPM12 (Wellcome Department of Imaging Neuroscience, University College London, United Kingdom), GIFT toolbox in MATLAB (http://mialab.mrn.org/software/gift/index.html) and the Advanced Normalization Tools (ANTs) (Avants et al., 2011) toolbox software packages. dMRI data were pre-processed using EDDY command adapted for

neonatal motion, from the neonatal dMRI automated pipeline from developing Human Connectome Project (dHCP, http://www.developingconnectome.org), and can be found at: https://git.fmrib.ox.ac.uk/matteob/dHCP_neo_dMRI_pipeline_release (Bastiani et al., 2019). Developmental RNA-seq data were accessed via: http://development.psychencode.org/.

Normalization	The GM-masked ICA-based brain atlas in the subject's structural space was registered to the subjects' dMRI space, as well as to the subjects' functional (BOLD) space using non-linear registration, diffeomorphic symmetric image normalization algorithm with cross-correlation as similarity metric (SyN-CC), from the Advanced Normalization Tools (ANTs) toolbox. (Avants et al., 2011)
Normalization template	The fMRI data, the preprocessing steps included realignment to the mean functional volume, adjusting for motion, co-registration to the time-point specific structural image, alignment in Montreal Neurological Institute (MNI) space, and normalization, in which all the scans are warped to our cohort 40 wGA template.
Noise and artifact removal	Regarding diffusion MRI data, data were preprocessed using MRtrix3 (version 3.0rc3, https://www.mrtrix.org) (Tournier et al., 2019) for denoising, bias field corrections and intensity normalization. FSL's TOPUP (Andersson et al., 2003, Smith et al., 2004) was used to estimate the off-resonance field, which was then used as input for FSL's EDDY function optimized for neonatal diffusion data, correcting for distortions induced by susceptibility and eddy currents, as well as by motion-induced signal dropout and intra-volume subject movement (Andersson et al., 2016, Andersson et al., 2017, Bastiani et al., 2019). Data were visually inspected to assure quality of motion artifacts correction.
Volume censoring	For the fMRI data, all volumes with a frame-wise displacement (Power, Mitra et al. 2014) greater than 0.5 mm or with a rate of BOLD signal changes across the entire brain (DVARS) greater than 3% were removed, along with the two previous and the two subsequent images. The remaining images were included for further analysis. A minimum of 50% of volumes remaining was set as a sufficient criteria for inclusion.

Statistical modeling & inference

Model type and settings	Regarding the MRI data analysis, longitudinal (from 33- to 40-wGA) and cross-sectional differences (at TEA, between VPT at 40-wGA and FT newborns) in both cortical BOLD variability and microstructural diffusivities were assessed with paired-samples t-test and independent samples t-test, respectively, with FDR correction for multiple comparisons.<br>For the gene expression data analysis, a linear mixed effects regression model (LMM) was used to identify significant changes in gene expression as a function of regional cluster (1 and 2) and time, with fixed effects of sex and RIN (RNA integrity number), including specimen ID as a random effect.
Effect(s) tested	See above.

Specify type of analysis: ☐ Whole brain ☒ ROI-based ☐ Both

Anatomical location(s)	We have used an atlas comprising the main resting-state networks generated from our cohort of infants, comprising both VPT at 33- and 40 wGA, as well as the FT infants, and combined into a brain cortical parcellation atlas.

Statistic type for inference<br>(See Eklund et al. 2016)	Region-wise (per resting.-state network).<br>BOLD-signal variability: For each RSN, voxel-wise time-series were averaged to produce a single regional time-course, which was then bandpass filtered (0.01–0.1 Hz). BOLD signal variability (BOLD SD) was calculated as the sample standard deviation of the regional time-course, yielding 9 BOLD SD values per subject per time-point.<br>Diffusion MRI: For each RSN, microstructural diffusion metrics (e.g., FA, MD, MK) were extracted in the same regions.
Correction	FDR correction was used for multiple comparisons (region-wise).

Models & analysis

n/a	Involved in the study
☐	☐ Functional and/or effective connectivity
☐	☐ Graph analysis
☐	☒ Multivariate modeling or predictive analysis

Functional and/or effective connectivity	Not applicable. BOLD signal variability was estimated by computing the sample standard deviation of the BOLD time-course within each of the 9 resting-state networks (RSNs).
Graph analysis	*Report the dependent variable and connectivity measure, specifying weighted graph or binarized graph, subject- or group-level, and the global and/or node summaries used (e.g. clustering coefficient, efficiency, etc.).*
Multivariate modeling and predictive analysis	For the gene expression data analysis, a linear mixed effects regression model (LMM) was used to identify significant changes in gene expression as a function of regional cluster (1 and 2) and time, with fixed effects of sex and RIN (RNA integrity number), including specimen ID as a random effect.
