# Supplementary material for: Regional BOLD variability reflects microstructural maturation and neuronal ensheathment in the preterm infant cortex
Source: Nat Commun. 2026 Apr 9;17:4849. doi: 10.1038/s41467-026-71415-x (PMC13222875; doi:10.1038/s41467-026-71415-x)
Supplement: Supplementary file 4 — Source Data [file 41467_2026_71415_MOESM4_ESM.zip › SourceData/README.rtf]

Regional BOLD variability reflects microstructural maturation and neuronal ensheathment in the preterm infant cortexSource code, READ ME fileJoana Sa de Almeida, 2026- Table 1: Individual participant values for all clinical and demographic variables are provided. Participants with analysable BOLD data are listed in “Table1.1_BOLD_clinicaldata.csv”, and participants with analysable diffusion data are listed in “Table1.2_Diffision_clinicaldata.csv”.- Figure 1: Boxplots showing individual subject BOLD SD changes from 33 wGA to TEA were generated from the “scaled” values from the file “Fig1.1_BOLD_data_subjects_scaled.csv”. Embedded group-averaged Bar plots illustrating the relative spatial distribution of longitudinal changes across cortical resting-state network regions were derived from the “scaled” regional means in “Fig1.2_BOLD_data_results_scaled.csv”.- Figure 2: Boxplots showing individual subject diffusion microstructural changes from 33 wGA to TEA, and embedded group-averaged bar plots, were generated for each diffusion metric using the “scaled” values from the csv files: Fig2.1 to Fig2.18.-Figure 3c: The Volcano plot was originated using data from “Fig3.1_enrichment_results_VolcanoPlot.csv”.- Figure 3d: Line graph was originated from “Fig3.2_sigGenes_lineGraph.csv”.- Figure 3e: Violin plots were originated from the file “Fig3.3_sigGenes_Violinplot.csv”.- Figure 4: Boxplots showing individual subject BOLD and diffusion microstructural values of VPT at TEA and FT newborns, as well as the embedded bar plots illustrating the difference between groups are originated from the csv files: Fig4.1 to Fig4.8.   - Figure S1: For generating the boxplots and embedded bar plots illustrated in S1, we have used the “scaled” values data from the csv files: S1.1 to S1.6.- Figure S2: Boxplots showing the raw individual values distribution of BOLD SD and diffusion microstructural metrics changes from 33 wGA to TEA were generated using the raw “value” from the “subjects” csv files from Fig.2.1 to Fig.2.18- Figure S3a: The line graph illustrating the elbow method results was generated from “S3.1_data_Elbow.csv”.- Figure S3b: The heatmap illustrating the consensus clustering results for k=3 was generated from “S3.2_data_ConsensusMatrix_k3.csv”.-   Figure S4:  Boxplots and embedded bar graphs showing the cross-sectional BOLD SD, ALFF and fALFF results are generated from the csv files S4.1 to S4.6.-  Table S5: Group mean averages (VPT and FT) for each metric, per region, are based on the individual subject values provided in the csv files TableS5.1 to TableS5.8.
